# Fine Mapping and Candidate Gene Prediction for White Immature Fruit Skin in Cucumber (*Cucumis sativus* L.)

**DOI:** 10.3390/ijms19051493

**Published:** 2018-05-17

**Authors:** Hong-Yu Tang, Xu Dong, Jian-Ke Wang, Jun-Hui Xia, Fei Xie, Yu Zhang, Xuan Yao, Yue-Jin Xu, Zheng-Jie Wan

**Affiliations:** 1 Key Laboratory of Horticultural Plant Biology, Ministry of Education, College of Horticulture and Forestry, Huazhong Agricultural University, Wuhan 430070, China; tanghongyu@webmail.hzau.edu.cn (H.-Y.T.); dongxushen@126.com (X.D.); xiajh@mail.hzau.edu.cn (J.-H.X.); xf507@webmail.hzau.edu.cn (F.X.); yzhang@webmail.hzau.edu.cn (Y.Z.); xyjho@mail.hzau.edu.cn (Y.-J.X.); 2 Zhejiang Wuwangnong Academy of Seeds Science Co., Ltd., Hangzhou 310020, China; sihaizi88@163.com; 3 Key Laboratory of Horticultural Plant Biology, Ministry of Education, College of Plant Science and Technology, Huazhong Agricultural University, Wuhan 430070, China; xuanyao@mail.hzau.edu.cn

**Keywords:** cucumber, a single recessive gene, white immature fruit skin color, qRT-PCR

## Abstract

In this study, a single recessive gene (designated *w*_0_) was identified to control the white immature fruit color. Genetic mapping with simple sequence repeats (SSR) markers located the *w_0_* gene in the distal region of cucumber chromosome 3 (Chr.3). Fine mapping was then conducted using the method of draft genome scaffold-assisted chromosome walking with 7304 F_2_ individuals, which allowed for the assignment of the gene locus to a 100.3 kb genomic DNA region with two flanking markers, Q138 and Q193. Thirteen candidate genes were predicted in the 100.3 kb region. Quantitative real-time polymerase chain reaction (qRT-PCR) analysis showed that the expression of the *Csa3G904140* gene, which encodes a two-component response regulator-like protein, was much higher in the immature fruit skin of the green parental line (Q1) than in the white parental line (H4). A coding sequence analysis suggested that a single-base insertion occurred at the ninth exon, resulting in a frameshift mutation in *Csa3G904140* of H4, and the mutation was consistent with the phenotype in 17 green/white germplasms. Therefore, *Csa3G904140* was taken as the likely candidate gene controlling the immature fruit color of cultivated cucumber. This study will contribute to the cloning of candidate genes and the development of white cucumber cultivars using marker-assisted breeding.

## 1. Introduction

Cucumber, *Cucumis sativus* L. (2*n* = 2*x* = 14), an important cucurbitaceous crop, is one of the top ten vegetables in the world. For most horticultural crops including cucumber, immature fruit skin color is a highly important external quality trait that influences consumer choice [1]. Since traditional breeding takes a long time to produce results, studying the inheritance of fruit skin color will facilitate marker-assisted selection and promote economic value.

There are many studies carried on pericarp color in horticultural plants. The genetic model of fruit color in bitter gourd was found to be a quantitative model through generation mean analysis; green is the dominant trait to white [2]. Gene mapping of pericarp color in wax gourd showed that the trait was controlled by one single gene; dark green color was dominant over yellow color, and the locus was located on chromosome 5 [3]. The fruit color of sweet pepper at the marketable mature stage was controlled by different nuclear genes: green vs. yellow was controlled by one completely dominant gene, yellow vs. white was controlled by two duplicate dominant genes, and green vs. white had differences in three genes [4]. The fruit color of a tomato consisted of the pericarp color and flesh color, while the genetics between them were completely independent [5]; studies showed that the pericarp color was controlled by one pair of nuclear genes, where transparent color was recessive to yellow color [6]. A quantitative trait locus (QTL) approach suggests that *PavMYB10* could be the major determinant of fruit skin in sweet cherries [7]. In 2016, *PavMYB10* was confirmed to be a reliable DNA molecular marker to select fruit skin color in sweet cherries [8].

The inheritance of pericarp color in cucumber has also been studied. Fine mapping of the pleiotropic locus B for orange mature fruit skin color in cucumber was conducted and identified a 50 kb region containing an R2R3-MYB transcription factor [9]. Studies on the uniform immature fruit color trait have shown that the mottled fruit color phenotype (U) was dominant over the uniform fruit color phenotype (u), and a single gene was responsible for phenotype segregation [10,11]. The *u* gene was fine mapped between the newly developed codominant SSR markers SSR10 and SSR27 at genetic distances of 0.8 and 0.5 cM, respectively [1]. The *w*_0_ white immature fruit skin color gene was located on Chr.3 at approximately 1150 kb in cucumber [12]. Wang et al. [13] studied the genetics of the white immature fruit color trait in cucumber and identified the molecular marker SSR15312, which was located on Chr.3. A premature stop codon, due to a single-base insertion, was found to be responsible for white immature fruit color in cucumber; the sole candidate gene, associated with chloroplast development and chlorophyll biosynthesis, was named *APRR2* [14]. 

In this study, genetic analysis of different F_2_ and backcross populations in cucumber revealed that one single dominant gene was associated with white immature fruit color in cucumber, and the locus was mapped to Chr.3. Subsequently, 1655 homozygotes derived from the 7304 F_2_ individuals from the cross of the Q1 × H4 hybrid were used for fine mapping of the white immature fruit color gene. The gene was mapped to a 100.3 kb region between markers Q138 and Q193 at a genetic distance of 0.03 and 0.03 cM, respectively. Quantitative real-time PCR analysis showed that the expression of the *Csa3G904140* gene was much higher in the immature fruit of Q1 than in H4. Furthermore, there was a frameshift mutation in *Csa3G904140*. Therefore, *Csa3G904140* was taken as one likely candidate gene. These findings will facilitate marker-assisted selection of the white immature fruit color trait in cucumber breeding and the cloning of the white immature fruit color gene.

## 2. Results

### 2.1. Genetic Analysis of the Immature Fruit Skin Color Trait

**Figure 1:**
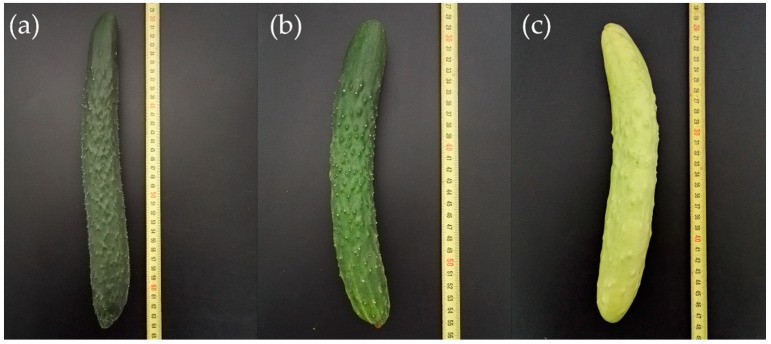
Immature fruit skin color of the parental lines and their progeny: (**a**) A green-skinned cucumber inbred line Q1 (P_1_); (**b**) A green-skinned F_1_ population of H4 × Q1; (**c**) A white-skinned line H4 (P_2_).

The skin color of two parental inbred lines and their offspring is shown in Figure 1, and the fruit skin color of the F_1_ cross (H4 × Q1) was green without segregation, revealing the recessive nature of the mutation. Genetic analysis of the immature fruit skin color trait is shown in Table 1. The F_2_ population derived from the crossing combination P_1_ × P_2_ segregated for 115 plants with green immature fruit skin and 29 plants with white immature fruit skin, which fitted an expected segregation ratio of 3:1 (*χ*^2^ = 1.56, *p* = 0.211). None of the BCP_1_ individuals from a backcross between F_1_ and P_1_ were white. For the backcross of F_1_ with P_2_, 65 plants had green fruits and 55 plants had white fruits in the BCP_2_ population, showing a segregation ratio of 1:1 (*χ*^2^ = 0.68, *p* = 0.411). These results indicated that the white immature fruit skin color in Q1 is controlled by a single recessive nuclear gene named *w*_0_.

### 2.2. Chlorophyll Content Determination and Chloroplast Observation

**Figure 2:**
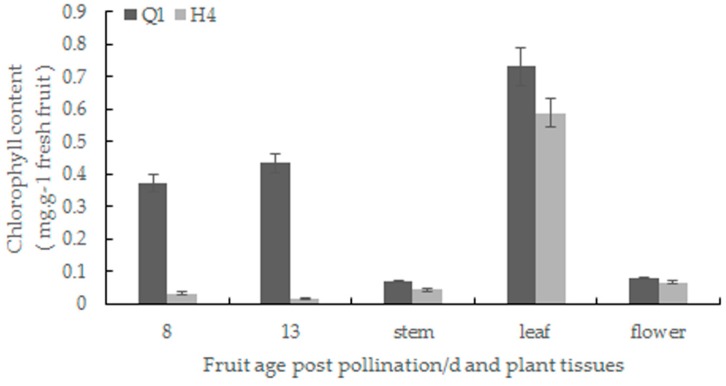
Chlorophyll content analysis in tissues between two parental inbred lines.

**Figure 3:**
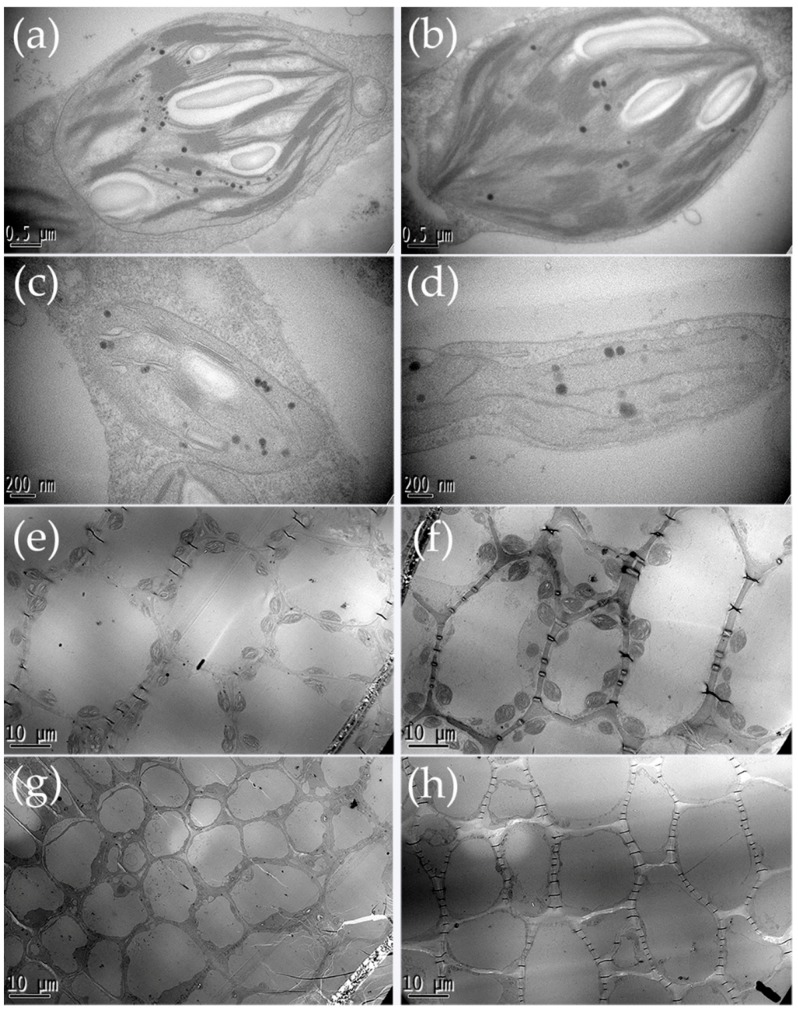
Epidermis chloroplast analysis of Q1 and H4 under microscopy: (**a**) epidermis chloroplast ultrastructure of Q1 in 8-day-old after pollination; (**b**) epidermis chloroplast ultrastructure of Q1 in 13-day-old after pollination; (**c**) epidermis chloroplast ultrastructure of H4 in 8-day-old after pollination; (**d**) epidermis chloroplast ultrastructure of H4 in 13-day-old after pollination; (**e**) epidermis chloroplast number and size of Q1 in 8-day-old after pollination; (**f**) epidermis chloroplast number and size of Q1 in 13-day-old after pollination; (**g**) epidermis chloroplast number and size of H4 in 8-day-old after pollination; (**h**) epidermis chloroplast number and size of H4 in 13-day-old after pollination.

High levels of chlorophyll were detected in the pericarp of 8- and 13-day-old fruit, stems, leaves and flowers in the green parental line compared to the white parental line, and the significant difference was found in the pericarp of fruit (Figure 2). Next, transmission electron microscopy was used to observe the number and ultrastructure of chloroplasts in the pericarp of the two parental lines. The chloroplast of H4 exhibited extensive internal vacuolization and premature senescence. The grana thylakoid of H4 lacked starch grains, and the internal structure of the plastid was fuzzy. The total number of plastoglobuli was decreased in H4 by comparing with Q1 (Figure 3a–d). Moreover, the chloroplast number and size of H4 were fewer and smaller than Q1 (Figure 3e–h).

### 2.3. Preliminary Mapping of the White Immature Fruit Skin Color Trait

To determine the chromosomal location of the *w*_0_ gene, 170 pairs of SSR primers (Table S1) were selected from the published 995 pairs of SSR markers in cucumber [15] at an interval of 3.5 cM. One polymorphic marker, SSR15312, was identified through bulked segregant analysis (BSA).

The SSR15312 was then used to genotype 144 F_2_ individuals, and this marker was found to be closely linked with fruit skin color. Moreover, the linkage between the marker and the phenotype was also detected in 120 progenies of the BCP_1_ population and 72 progenies of the BCP_2_ population. These data proved that the marker SSR15312 was closely linked to the target gene. Ren et al. [15] reported that SSR15312 is located on Chr.3, suggesting that the *w*_0_ gene is mapped to Chr.3.

### 2.4. Fine Mapping of the White Immature Fruit Skin Color Trait

**Figure 4:**
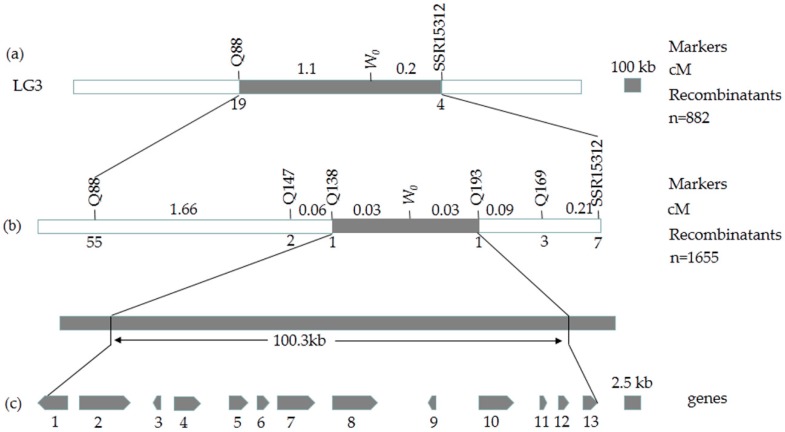
Sketch map of the fruit skin color gene and candidate gene analysis: (**a**) Linkage map constructed using 882 F_2_ recessive individuals. The *w*_0_ gene was mapped to the region between markers Q88 and SSR15312; (**b**) Fine mapping of the *w*_0_ gene locus. The *w*_0_ gene was localized to the region between the flanking makers Q138 and Q193 using 1655 recessive individuals; (**c**) The annotated gene in the candidate region of the *w*_0_ gene locus.

To fine map this gene, an F_1_ population of H4 × Q1 was self-pollinated to produce an F_2_ population, 175 pairs of cleaved amplified polymorphic sequences (CAPS) markers (Table S2) were developed with 3916 F_2_ individuals. Among them, four pairs of polymorphic markers, Q88, Q138, Q139, and Q4-2, were identified and genotyped in 882 white plants in the F_2_ population. Q88 was at the left side of SSR15312 with 19 recombinants, while SSR15312 had 4 recombinants. In addition, the markers, Q138, Q139 and Q4-2, co-segregated with the target gene (Figure 4a). Then, the F_2_ population was enlarged to 7304 individuals, another 63 pairs of CAPS markers (Table S2) were developed, including three new polymorphic markers, Q147, Q193, and Q169, were developed.

Linkage analysis of these eight pairs of polymorphic markers with the *w*_0_ gene of cucumber using 1655 white plants in the F_2_ population verified the recombination rate to calculate the genetic distance. The results indicated that there was still no recombination between Q139, Q4-2, Q142 and the white immature fruit skin color gene, while two different recombinants were detected between the two closest flanking markers Q138 and Q193 at a genetic distance of 0.03 cM (Figure 4b).

The two closest flanking markers, Q138 and Q193, were developed from the *Csa3G904060* gene and the *Csa3G910680* gene. A basic local alignment search tool (BLAST) of the cucumber genome sequence was used (http://cucurbitgenomics.org/blast) to determine that two genes are located at positions 39193398 (the first base of the 5′-terminus sequence) and 39293751 (the last base of the 3′-terminus sequence) on Chr.3, respectively; the physical distance between them is 100.3 kb, which contains 13 candidate genes (Figure 4c).

### 2.5. Candidate Gene Prediction and Gene Expression Analysis

**Figure 5:**
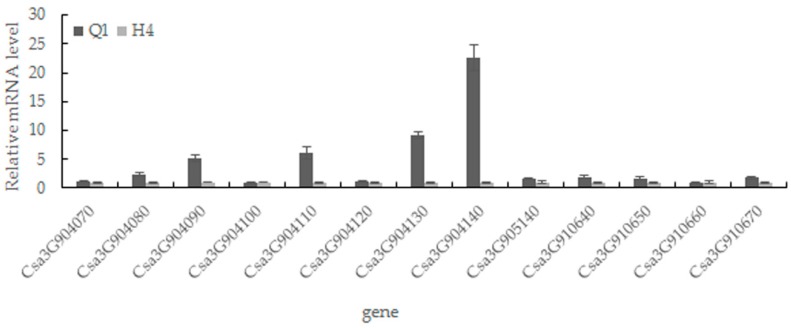
Quantitative real-time PCR analysis of the candidate gene in Q1 and H4. The data are presented as the average values of three replicates.

**Figure 6:**
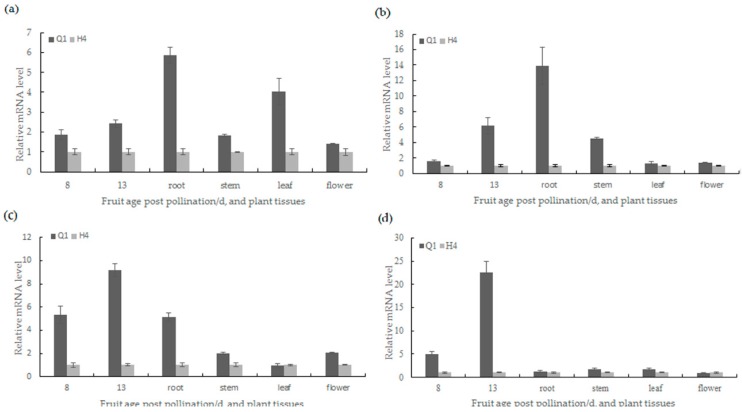
Quantitative real-time PCR analysis of candidate gene in tissues between Q1 and H4: (**a**) relative mRNA levels of the *Csa3G904080* gene in different tissues; (**b**) relative mRNA levels of the *Csa3G904110* gene in different tissues; (**c**) relative mRNA levels of the *Csa3G904130* gene in different tissues; (**d**) relative mRNA levels of the *Csa3G904140* gene in different tissues.

The function of all 13 candidate genes was predicted by Cucurbit Genomics Database (http://cucurbitgenomics.org/search/genome/2) (Table 2). Then, qRT-PCR analysis (primer sequences are available in Table S3) was performed to test whether the gene expression was altered between the two parents. As shown in Figure 5, the expression levels of the *Csa3G904130* and *Csa3G904140* were 9.2-fold and 22.6-fold higher in Q1 than H4, respectively. The other two genes (*Csa3G904080* and *Csa3G904110*) were 2.4-fold and 6.2-fold higher in Q1 than H4 in expression quantity. The results of the tissue expression revealed that *Csa3G904080* was highly expressed in root and leaf; *Csa3G904110* was highly expressed in pericarp, root and stem; *Csa3G904130* was highly expressed in pericarp and root; While *Csa3G904140* was highly expressed in the pericarp of fruit rather than other tissues (Figure 6).

**Figure 7:**
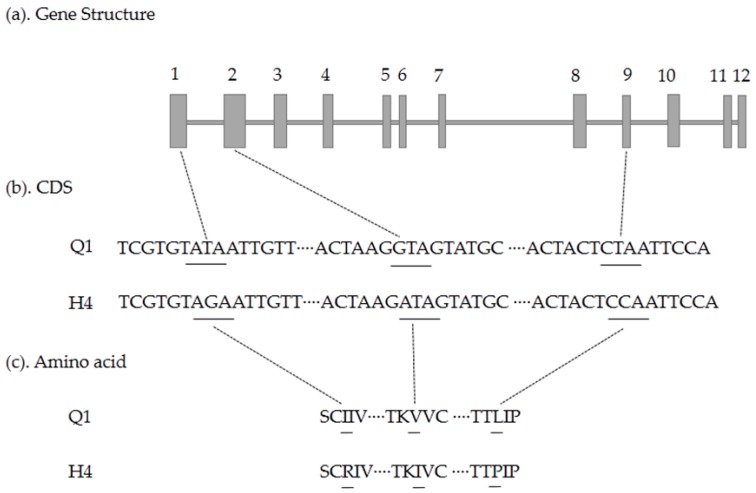
Comparison analysis of the cDNA sequence and amino acid sequence of the *Csa3G904080* gene between Q1 and H4; a 3-bp mutation resulted in three amino acid mutations: (**a**) gene structure of *Csa3G904080*, including twelve exons and eleven introns; (**b**) coding sequence analysis of three SNP mutations; (**c**) three amino acids mutation due to three SNP mutations.

**Figure 8:**
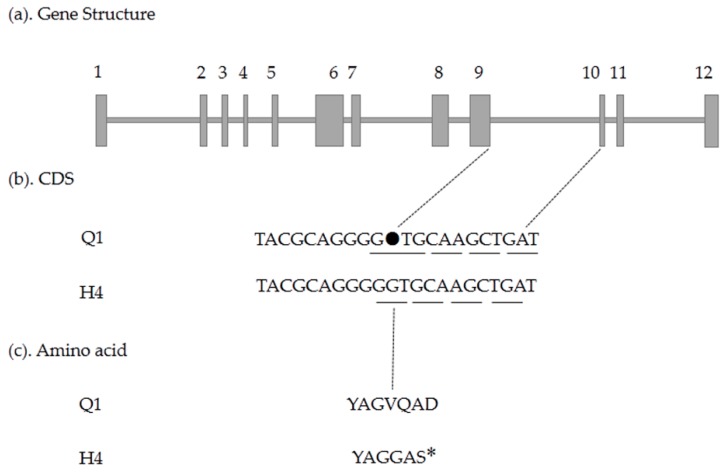
Comparison analysis of the cDNA sequence of *Csa3G904140* gene between Q1 and H4; the insertion lead to a premature stop codon: (**a**) gene structure of *Csa3G904140*, including 12 exons and 11 introns; (**b**) coding sequence analysis of a single-nucleotide insertion; (**c**) a frameshift result in a premature stop codon; * delegates the stop of protein translation.

To analyze gene sequences of these four candidate genes, we designed primers (Table S4) to amplify the entire coding sequence and performed TA cloning. By comparing candidate gene sequences between the two parents, three single nucleotide polymorphism (SNP) mutations in exon were detected within the *Csa3G90408*0 gene, which led to three amino acid changes (I83R, V316I, and L1328P) (Figure 7); there was one single-nucleotide insertion resulting in a premature stop codon within the *Csa3G904140* gene (Figure 8), and two same sense mutations within the *Csa3G904110* gene. The cDNA sequence of *Csa3G904130* in Q1 was the same as that of H4. In addition, the mutation in the *Csa3G904140* gene was consistent with the phenotype of 17 green/white germplasms (Tables S5 and S6), while the mutations in the *Csa3G904080* gene were not consistent with the phenotype of 17 green/white germplasms (Table S7). Furthermore, the marker Q142 associated with the *Csa3G904140* gene cosegregated with white fruit skin color. Therefore, *Csa3G904140* was likely to be the candidate gene controlling the white fruit skin color in cucumber.

## 3. Discussion

The white immature fruit skin color is one of the most valuable external quality traits in cucumber, which may directly affect a consumer’s choice of cucumber fruit. Therefore, it is valuable to identify the genes or loci associated with white skin color. In this study, a recessive gene *w_0_* was identified to control white fruit skin color in cultivated cucumber, consistent with the results reported in previous studies [13]. Map-based cloning is a primary strategy that has been used to identify important trait genes in many organisms [16,17]. However, cucumber cultivars have a low genetic diversity, which increases the difficulty in the use of map-based cloning in cucumber [18]. Whole genome sequencing of the cucumber has enabled large-scale development of molecular markers for this crop [19]. Therefore, based on the published cucumber genome sequence (http://cucurbitgenomics.org/organism/2), markers were developed. By the stepwise increase of the mapping population sizes and taking advantage of draft genome assemblies, we identified the *w_0_* gene to a 100.3 kb region containing 13 candidate genes, which laid a solid foundation for gene cloning.

To ensure the accuracy of the result, we used the cucumber genome browser, version 2 (http://cucurbitgenomics.org/JBrowse/) and BLAST of the National Center for Biotechnology Information (NCBI) (http://blast.ncbi.nlm.nih.gov/Blast.cgi) to predict candidate genes in the target region. Consequently, a total of 13 candidate genes were identified in the region between the two markers Q138 and Q193. The results from sequence alignment analysis showed that *Csa3G904080* gene sequence between Q1 and H4 existed three amino acid mutations, but the mutations were not consistent with the phenotype of 17 green/white germplasms. In addition, the gene expression of *Csa3G904080* was higher in root and leaf than in fruit skin, and the gene function is related to photosynthesis. Thus, it was unlikely that *Csa3G904080* was responsible for the white pigmentation. C*sa3G904110* and *Csa3G904130* had a high relative expression level in fruit skin, however, *Csa3G904110* had a more high expression level in the root and *Csa3G904130* also had high expression in the root, and these two genes had no amino acid mutation between Q1 and H4. For another nine candidate genes, the relative expression level in fruit skin was too low. While the *Csa3G904140* gene contained one single-base insertion between the two parental inbred lines, and the mutation was consistent with the phenotype of 17 green/white germplasms. Moreover, a 22-fold increase in the expression of *Csa3G904140* was found in the green-skinned parental line Q1. Therefore, the *Csa3G904140* gene was likely to be the candidate gene controlling immature fruit skin colors of cultivated cucumber. 

The chloroplast is a half-autonomous organelle that commonly exists in land plants, algae and protists and is responsible for photosynthesis. A search for conserved domains against NCBI CDD (http://www.ncbi.nlm.nih.gov/structure/cdd/wrpsb.cgi) revealed that *Csa3G904140* contained a MYB-like DNA-binding domain. This DNA-binding domain is primarily responsible for the color of fruit, many of which also contain a transcriptional activation domain [20,21]. Studies have shown that nuclear *Sig* genes—encoded sigma factors—play a key role in controlling RNA polymerase binding to promoters in chloroplasts [22]. Nuclear-encoded proteins greatly influence chloroplast RNA editing, post-transcriptional processing and the maintenance of RNA stability [23]. Subcellular localization of the Csa3G904140 protein was predicted using Plant-PLoc (http://www.csbio.sjtu.edu.cn/bioinf/plant/#) to show its presence in the nucleus. 

Pan et al. reported that an *APRR2* gene increased the pigment content and the levels of chlorophyll in immature fruit [24]. Moreover, *APRR1*, which is related to *APRR2*, was thought to be involved in plastid development and ripening [24,25]. A single candidate gene, *APRR2*, was defined to control white immature fruit skin color in cucumber by delimiting the physical interval to an 8.2 kb region [14]. The NCBI annotation of *Csa3G904140* was predicted to be *Cucumis sativus* two-component response regulator-like *APRR2*. In our study, the chlorophyll content was much higher in the green compared to the white lines, and microscopy revealed that the chloroplast number and size of H4 was less and smaller than Q1. This gene, *Csa3G904140*, seemed to be the candidate gene for the proposed gene locus conditioning immature fruit skin colors of cultivated cucumber. Whether this *w_0_* gene was the same as in a previous report [14] requires further study.

## 4. Materials and Methods

### 4.1. Plant Materials and Genetic Mapping Population

A green-skinned cucumber inbred line Q1 and a white-skinned line H4 were used as the male and female parent, respectively, to generate one F_1_ population, one F_2_ population and two BC_1_ populations, including BCP_1_ and BCP_2_. Their skin colors were uniform, and both lines were from our research group. In these 17 green/white cucumber lines, some were obtained from the National Vegetable Germplasm Resources Intermediate Library of the National Crop Germplasm Resource Platform—Vegetable Germplasm Resources sub-platform (China), and other germplasms were provided by our research group.

Genomic DNA of all individuals was isolated from young leaves using a modified Hexadecyltrimethy Ammoniun Bromide (CTAB) method described by Porebski et al. [26]. Six DNA samples of green and white skin phenotypes were bulked and used to identify markers linked to the color gene; the method was described by Michelmore et al. [27]. All experiments were conducted during the growing seasons between 2012 and 2017 in the National Vegetable Improvement Center and the State Key Laboratory of Horticultural Plant of Huazhong Agricultural University.

### 4.2. Chlorophyll Content Determination and Chloroplast Observation

Pericarp samples from 8- and 13-day-old fruits after pollination and other tissues (roots, stems, leaves and flowers) were excised and extracted with 96% alcohol in the dark. After 24 h, the extracted pigments were immediately spectrophotometrically determined at specific absorption coefficients using the method described by Wellburn et al. [28].

To observe the chloroplast ultrastructure of the pericarp, tissues from 8- and 13-day-old cucumber fruit (after pollination) were excised with a sterile razor blade and fixed immediately in 3.5% (*v*/*v*) glutaraldehyde solution. Chloroplast observations were conducted with a H7650 microscope (HITACHI, Tokyo, Japan).

### 4.3. Design of SSR and CAPS Markers

Sequences of the SSR primers were derived from the Cucurbit Genomics Database (http://cucurbitgenomics.org/). A total of 170 pairs of SSR primers distributed evenly on seven chromosomes were synthesized by Sangon Biotech Co., Ltd. (Shanghai, China).

PCR amplification for SSR analysis was carried out in a 10 μL volume containing 1 μL (50 ng/μL) template DNA, 1 μL primers (10 μM), 5 μL 2× Taq PCR Master Mix and 3 μL ddH_2_O. The reaction conditions were as follows: 94 °C for 5 min, followed by 35 cycles at 94 °C for 30 s, 56 °C for 30 s, and 72 °C for 30 s, with a final extension at 72 °C for 10 min. The amplicons were separated on 9% denatured polyacrylamide gels [29].

CAPS primers were developed using Primer 3.0 (http://bioinfo.ut.ee/primer3-0.4.0/) according to the cleavage sites of genome sequence (http://cucurbitgenomics.org/organism/2) in the candidate region, and synthesized by Sangon Biotech Co., Ltd. (Shanghai, China). The PCR for CAPS was also carried out in a 10 μL mixture, similar to that for SSR markers. The reaction conditions were as follows: 95 °C for 4 min, followed by 30 cycles at 94 °C for 30 s, 56 °C for 30 s, and 72 °C for 1 min, and a final extension at 72 °C for 7 min. The amplicons were separated on 9% denatured polyacrylamide gels. Non-polymorphic amplicons were digested with restriction endonucleases. A 10 μL digestion system included 4 μL PCR product, 0.1 μL enzyme, 1 μL buffer, and 4.9 μL ddH_2_O. The digestion process was adjusted according to the manufacturer’s recommendations for the corresponding enzyme. Polymorphic markers were used to genotype the population.

### 4.4. Gene Prediction and Expression Analysis

Two software tools, the cucumber genome browser, version 2 (http://cucurbitgenomics.org/JBrowse/) and BLAST of NCBI (http://blast.ncbi.nlm.nih.gov/Blast.cgi), were applied to predict candidate genes.

Quantitative real-time PCR was used to analyze the expression of the candidate gene in the two parents. Pericarp samples from 8-day-old and 13-day-old fruit after pollination and other tissues, including roots, stems, leaves, and flowers, were excised, and RNA was isolated using a modified Trizol method described by Cheng [30]. First-strand cDNA was synthesized with a Prime Script™ RT reagent Kit with gDNA Eraser (Perfect Real Time) (TaKaRa, Tokyo, Japan) according to the manufacturer’s instructions. The *18sRNA* gene was used as the reference, and the primer sequences are shown as follows.

18sRNA-F: AGAAACGGCTACCACATC
18sRNA-R: CCAAGGTCCAACTACGAG

Using a 96-well plate and SYBR Green-based dye, the qRT-PCR reaction system contained 1 μL template cDNA (100 ng/μL), 5 μL 2× SYBR Green PCR Master Mix (TaKaRa, Tokyo, Japan), and 0.4 μL forward and reverse primers (10 μM), with water added to a total reaction of 10 μL. The qRT-PCR was performed using a CFX96 Real-Time PCR Detection System (Bio-Rad, Hercules, CA, USA). The values from triplicate reactions were averaged, and the relative expression level was measured by means of 2^−ΔΔ*C*t^ [31].

### 4.5. Candidate Gene Cloning and Sequencing

TA cloning was used to analyze the sequence of the candidate gene in the two parents. RNA was isolated using a modified Trizol method as described previously [30]. First-strand cDNA was synthesized with a Prime Script™ II 1st Strand cDNA Synthesis Kit (TaKaRa, Tokyo, Japan) according to the manufacturer’s instructions. Primers were designed to amplify the entire coding sequence. PCR amplification was carried out according to the user manual with I-5™ 2× High-Fidelity Master Mix (MCLAB, San Francisco, CA, USA). The amplicon was separated on 1% agarose gels and extracted with a TaKaRa MiniBEST Agarose Gel DNA Extraction Kit Ver.4.0 (TaKaRa, Tokyo, Japan) according to the manufacturer’s instructions. The purified DNA product was then subjected to a “+A base” reaction using a kit (ZOMANBIO, Beijing, China), ligated to the pMD18-T vector with a pMD™18-T Vector Cloning Kit (TaKaRa, Tokyo, Japan), and inserted into DH5α chemically competent cell (Weidibio, Shanghai, China) according to the manufacturer’s recommendations. Finally, three correct monoclonal spots were sequenced by Sangon Biotech Co., Ltd. (Shanghai, China), and the sequences of the two parents were compared using the DNAMAN program.

## Figures and Tables

**Table 1:** Segregation of immature fruit color in the three populations of cucumber.

Population	Observation		Expected Separation Ratio	*χ*^2^	*p* Value
	Green	White			
BCP_1_^a^	120	0	1:0	-	-
BCP_2_^b^	65	55	1:1	0.68	0.411
F_2_^c^	115	29	3:1	1.56	0.211

^a^ BCP_1_ = F_1_ (H4 × Q1) × Q1; ^b^ BCP_2_ = F_1_ (H4 × Q1) × H4; ^c^ F_2_ population was derived from the self-pollination of F_1_ (H4 × Q1).

**Table 2:** The Cucurbit Genomics Database description of all 13 candidate genes.

Gene ID	Cucurbit Genomics Database Description
*Csa3G904070*	Putative peptide/nitrate transporter; contains IPR000109 (Proton-dependent oligopeptide transporter family), IPR016196 (Major facilitator superfamily domain, general substrate transporter)
*Csa3G904080*	Pyruvate kinase; contains IPR001697 (Pyruvate kinase)
*Csa3G904090*	Unknown protein; contains IPR008502 (Prolamin-like domain)
*Csa3G904100*	Ribose-phosphate pyrophosphokinase; contains IPR005946 (Ribose-phosphate diphosphokinase)
*Csa3G904110*	Tobamovirus multiplication 2B
*Csa3G904120*	Peroxidase; contains IPR010255 (Haem peroxidase)
*Csa3G904130*	Tetraspanin family protein; contains IPR018499 (Tetraspanin/Peripherin)
*Csa3G904140*	Two-component response regulator-like protein; contains IPR009057 (Homeodomain-like), IPR011006 (CheY-like superfamily)
*Csa3G905140*	Unknown protein
*Csa3G910640*	Unknown protein; contains IPR018996 (Inner nuclear membrane protein MAN1)
*Csa3G910650*	Allyl alcohol dehydrogenase-like protein
*Csa3G910660*	DnaJ homolog subfamily C member; contains IPR009057 (Homeodomain-like)
*Csa3G910670*	Expansin L; contains IPR007117 (Expansin, cellulose-binding-like domain), IPR014733 (Barwin-like endoglucanase)

## Supplementary Materials

Supplementary materials can be found at http://www.mdpi.com/1422-0067/19/5/1493/s1.
