# Supplementary material for: Fine Mapping and Candidate Gene Prediction for White Immature Fruit Skin in Cucumber (Cucumis sativus L.)
Source: Int J Mol Sci. 2018 May 17;19(5):1493. doi: 10.3390/ijms19051493 (PMC5983590; doi:10.3390/ijms19051493)
Supplement: Supplementary file 1 [file ijms-19-01493-s001.pdf]

**Table S1.** The sequence of SSR primers used in preliminary mapping of the white immature fruit skin color trait

| Name | Forward Primer Sequence | Reverse Primer Sequence |
|---|---|---|
| SSR23757 | TTCGCCTTTACTTCCTTCCA | CGGCAAAATTCCTCCATTTA |
| SSR05501 | GGAATTATGCAATTTGGGCA | AACCAAACAATGCCCTAGCA |
| SSR02288 | TGAAGTCGATGGAATCTTTTGA | TGGGGAAAGAGCTAAGAAGAAA |
| SSR22638 | TGTGTAAGATTTTTATTGGATGCC | CTGAGCTTGATCAATTCCTTCA |
| SSR12573 | CGAAGATCAAATCGCAAACA | TGCTGGTCCTCGTCTTCTTT |
| SSR16621 | CAAAATTTGAAGACTAAATTGGCT | TGCTTTGATTTCATTTGCCA |
| SSR00991 | TCCCGTTTGATTTCACTGCT | CCTGAATCAGCAACAGACGA |
| SSR21663 | ATCCCCATAATCCCATTTCC | AGCATTATGAATGGAGGGCA |
| SSR10188 | GGTCCTTTTGCAGTGAGCTT | GCAAACATGTCAACTGTCCG |
| SSR32126 | ATCCTAGAATGCACGAACGG | AAGGTTGAAAGTATAACAAAACAATG |
| SSR12032 | GGGGGTTTGAATTGAATGTG | CTCTTCTTTCCCCCTTTGCT |
| SSR03066 | CAAAACTTAAGGACCGAAAGGA | ACATGGTTGGTTAGTGGCCT |
| SSR01601 | CTCTCTTTGTTGGCACCCTC | ACCAAGCGAACTAGAGAGCG |
| SSR19565 | ACACCCCGCTCTAGGTTTTT | CCATACAAGGGTGGAATTGTG |
| SSR02697 | TGCTAACCCAACCAAACAAA | CTGCCATTTCAAGCTATGGG |
| SSR22862 | AATTGGGTCAAAGAAAGGGG | TCGTCAAAATGAATCTCCGA |
| SSR11452 | AATCAAAATTAGCACATTAGGATAAAA | TTGCAAGGGTTGAGTACCATT |
| SSR02244 | GATCGTAGGATTGATTGGCA | TGGCACCAATTCAGATACCA |
| SSR17975 | GAGGACAACTCTGCTACGGC | CACCTCCATAGCCAGAAACC |
| SSR12502 | ACTCCTCTTTCATGCTCCCC | GTACGTGTGTACGAGCATGTG |
| SSR02384 | AAAAATCCGACAAATCGTGC | GGTCAAATGTTGCCTTTTGC |
| SSR18780 | CCCATCATGTGATCTACTCAGAA | AAATGAAAAACAAATGAAAATAGGA |
| SSR19842 | CCCCACCTAAACCTTTCCAT | GTTTGTGCAAAACCCAATCC |
| SSR03150 | TTGATTGAATGAATGGTTTGGA | TCAAAATTGATTGGTAAGAAGAAGC |
| SSR05978 | AGTCCACCCTCAAATGCAAC | CATACCATTCCACCGTAGGAC |
| SSR11742 | GCTATCCCCAAGGATGATGA | AGCTTGGCTTCGTCTTTTGA |
| SSR22593 | TCTGAGTTCCATCCCCTTTG | CAATGTGAGTTTTTGGTTGAGA |
| SSR19844 | ACCCATCAACCCATCAACAT | TGGAAGTTGAAAAATGGGAA |
| SSR12258 | GGTTCAAATTTCAAAGTGTATGAAT | GCCAAAGCTTCATAGTGAGCA |
| SSR05695 | CACTCTTGTGGAATCCTTCCT | AGAACCAAGACGGCAATGAC |
| SSR03462 | CACGCGAGAGGAGAGGATAG | GAAACAAAATCTGGGGTCCA |
| SSR16130 | TGCCACAGGGATTCCTTCTA | TGAAACCCATTTGAGATCTCTTC |
| SSR10018 | CTTTTGTTCTTGTGGAATGTGA | ATTTGGGGATGGAGAGGTTC |
| SSR16869 | GAAAACATGAAGGCCGTTGT | CCATTTGTCAGTGCCTTTCA |
| SSR20162 | TGCCTTCCCATATCAAAACA | GGTCATTGTCAGGAAACAAAAA |
| SSR00168 | TAAGCATTCCCCACTTTTGG | GGAAGACACGAGAGAGACGG |
| SSR06755 | GTGGGTGGATGGATTTGAAC | GGAAAATGGACCAAAGATAGACA |
| SSR20473 | ATGTTCAGGGCCTGGTATTG | TCCAACAGCTCCAATTAGCC |
| SSR06576 | TGATCATGGGAAGAGAGAGACA | TCAAGAAATGTGATGAATGGAAA |
| SSR24036 | AAGCTAAATTTATGAAATGAATGACG | TCATAAGTTAGGATTTGCACTTATTTT |

| Name | Forward Primer Sequence | Reverse Primer Sequence |
| --- | --- | --- |
| SSR13150 | TGTTTTGAAAATTTTGGTGTGA | TTTCGGAATGAAAATCGTTAAA |
| SSR23420 | GAAGGGATGGTAGATGAAGGG | CTTCTCCCCTTTCTTGGCTT |
| SSR20248 | TCCTCAAACGTCTCTCTCCC | GATAGCATGCGGTGTCCTCT |
| SSR02539 | AAAAATGATCAGCTCGATGAAA | GCAAGCGCTTTCCAATCTAT |
| SSR10522 | TTCCTTTTGTTTTTGGTATGGG | ATGTCTGCTTTGCTGGCTTT |
| SSR20932 | TGAAACCAAAGTCCCACTCC | TCGACCTCTCTCTCACACACA |
| SSR20045 | TGCACATGTGTAAGGCTTTG | AGGGTGGAGGAATACATTGAA |
| SSR16183 | GGAGAAATTTGATGGTGTAGCC | TTGCAAATCTCTAATACTTTGCCTT |
| SSR11908 | TTTCCAATTGATATCTTCTGCATT | TTTAAAACACGTCGCTAATCTATCA |
| SSR14841 | AACAGATCTTGTTTGCATGGG | TGGACCCAAGAGTTCCTCAC |
| SSR20251 | ATTTGACCTCCCTCTCTCCC | TCATTGCTCAAACCTTTTCG |
| SSR01981 | GTTTGGACGACTGTGTGGTG | GGGAGCATCTCCTCTTAGATATG |
| SSR18640 | GGCGTTGGGTTAAGCATTTA | CGTGGGTTTTTACCGTCATT |
| SSR13274 | TCCACTCAAACTTTCTCCCC | CAACATTGGATGAGCTTTGC |
| SSR11633 | AATGAGATAACATATGGATTTGTATGA | CACCGAACTGAAGTGTGCAG |
| SSR18613 | CCACATAAAACAGCAAGGCA | TGTTGGTTGGTTGACTAGCATT |
| SSR07505 | GACAGGACCGTTAACCCAAA | CTCCCTCTTTCCCTCACTCC |
| SSR23574 | CAGTTCACAACCACATCTTTGAA | CCGAGCTAACAACCCAATCT |
| SSR15124 | TCCACCCAATCTTTCCACTC | AGTGCTCTTCACATTTGCCA |
| SSR22231 | CATGTGTGACGCTTCTTTATTTTT | TGGTGCTCCCTTTCTACACA |
| SSR17024 | GATGTCACCGAACCAAGTGA | CCCACAAAGAAGGTTTCAGA |
| SSR16936 | TAGGCCGAGTTGGTGCTAGT | GCTGCATTTGGAATCCTTGT |
| SSR04385 | ATGTGGGGTTAAAAATGGCA | ATGCCACGTACCTGTCACAA |
| SSR02803 | ATTGCTCCCAAGCAACTTGA | ATTTCAAACCTCCAAGGCTG |
| SSR21197 | AAACCATTTGGTATTGGCGA | GTCCAACCAAACCAAGCCTA |
| SSR00203 | AATAGCTCGAAAATGATGGCA | CCTCAAAGAGGATCAAGCGA |
| SSR31064 | TGCCAAAATATGAGTTTAGCCC | GGCCATTGAAGGTGAATTTG |
| SSR01610 | TGGTTGTATATAGAAGGAAGATGTTGA | CCCTTTGAACGAAATCCAAA |
| SSR00023 | GCACGGATTCTGGGTTTAGA | TCATCCTTCGGAATCGTACA |
| SSR05513 | TCTTCTTATTTGCATCCGGC | CTCGCTTTCTCTCGAGCTGT |
| SSR04836 | GACAGTCTGACAGTTCCCCAA | GAGCATCTAGCTGGGGTCAC |
| SSR12291 | CGCACGAGAACCTTTATTGA | TCACATCAAATTAACACTTTCATCTC |
| SSR16308 | CGAAACAAAAGTTTATGATTTTATCG | TTTTTAAAACAATCAATACATGTCAAG |
| SSR14608 | CCAACACCTGGGTTTGCTT | AAAGAAGACGAAGAGGAAGAGGA |
| SSR19538 | TGCATGCAAAATAGGCAAGA | GCCCTGCAGTTTCTTCAGTC |
| SSR00170 | TTGCAATTTGTGCAGGGATA | AGGTATTATGGCCCAAAGGG |
| SSR21219 | CCATTTCAATCCGCATAACC | CGCAATTGACGGCTATGTTA |
| SSR18949 | CTATGGGATCTGGCTCTGGA | GGGTTCACGCCAGTATTGTT |
| SSR14084 | GGGTATTTGTCCAATTTCCTAATG | TTTGTGTGGTACCCCAAGTAAT |
| SSR16020 | ACACCATTTTTCATCGAGATTT | GGGATGAGGAGCAAATGGTA |
| SSR30353 | GGACCATTAACTTCCACCCC | ATGGGATTTGCCTGTAATGC |
| SSR00842 | CGCCCAAATTGAACGAATAA | CCTCCGCCTTTCTTTCTTTT |
| SSR28064 | GAAATTAATTAAGATCAAGAAAGGGA | ATCACTTGCATCACGTCTGG |

| Name | Forward Primer Sequence | Reverse Primer Sequence |
|---|---|---|
| SSR05348 | ACTGCTGGAAAAAGCTGGTG | AGCCTATTTCCCCGCTCTTA |
| SSR21936 | TTGGTTGGAAAAAGGAAGGTT | GGGCAGAGGCTTTTTCAATA |
| SSR06240 | TTGAACATGAAAAGTATTGGCG | TTGCAACTAAGGTGTGCTATTCTC |
| SSR04314 | CCATGGGGTTCTGTAGCTGT | TGCAGGTGTGGAGAAAAGAA |
| SSR04689 | TCTCCGGCAGAAAGAAAAGA | TGCGTCTCCTTCTTCCTCAT |
| SSR00084 | AAGTGTCAGCATGTCGTTCG | AAACGTCAGCCACACCTTTC |
| SSR00218 | CGATCTTCGAGTTTCGCAAT | ATCCAACGGCTCTCATTCAC |
| SSR01099 | TTTCCTAGCCAAATCATGCC | CTCGGGTCAGCATCTTTTTC |
| SSR00048 | CCGATCGTTGCTAGGAGAAC | CTCCCGAAACCTAGAAACCC |
| SSR17352 | TCATCTAAATATAAAGTTGAATGGTTG | CTCCTCACTCACACTTCGCC |
| SSR15006 | CACACAAGGTTTTAGCCACCT | TTGCCATCTTATCTCACAAGG |
| SSR00477 | TATTGCGATGGTTTGACGTG | GCAAATTCCGGAGTTCGTTA |
| SSR15110 | ACGCGATTGAGCCTTCTAAA | TTGAAGATGACGAGCAAAAGA |
| SSR03070 | GCTAACACTACCCGCTGCTT | AACAGAAAGAGAATCGGGGG |
| SSR12124 | ACCTAAGGTTAAGGCGTGATAA | TGGTTGTTGATGTGACTTAGCC |
| SSR18937 | TTACTCCAAAGATGCTGGGC | CATTTGACCGAATCTTGACTTT |
| SSR00387 | TGACTTCCGACTTATGTGGATT | CTTTCTATGGCACGCATTCA |
| SSR22558 | TCAACTTATCCCTCTTTCTATTTTCC | GGCAATCATTACCAAAAACCA |
| SSR21570 | ATGTGAAGAAGCAGATGAAAATTA | TTTGTGAAGGTGCATTTTATCG |
| SSR10849 | TCGATCTGGTTTACTGTAGATATGC | GAAGAATTTGAAAAGATAAAAACACA |
| SSR00378 | TCCCTAAAATTTCGACAACCC | TTAGTATGGCTTGAACACCCA |
| SSR00030 | TGAAATTGCTTACCCTTTGACC | CCATGTTTTGTAGGGATCGAG |
| SSR24696 | TCAAGTCAAGAGCTTGATGACAA | CAACAGTTTAGTTGGAAAGTGACAA |
| SSR20268 | GCAGGATATATTTCGAAAACTGAAA | AAGTGAATTGCTAACAAGAAACG |
| SSR04219 | GAGACATTGTGGGCATTTGA | CTCATTTTCATCCAAAGGGC |
| SSR22832 | TTTGGACTAGCTACGGAACAG | CATCTCAATTTGGAACCTCCAT |
| SSR22832 | TCACATAGGCTTGCTCCAAA | TCAAACACCGCGAAAGAGAT |
| SSR02634 | GGGTTTGTGACACGTTTCCT | GGCAAAGGCAACAAGTTTCT |
| SSR15312 | CCCGCTTCTTCTTCCTTCTT | ACTTCTCCGGGAAAATCCAC |
| SSR16135 | GCTAAGTTATTGTTGACCTCAACTTCT | CGTGTGAAATTCAACCTATTCTCTT |
| SSR16916 | AGCATGATGAGGATCCCTTG | CCGAACTGCACAAAGTATGG |
| SSR21502 | CGGGTCAATTCCATATATTACAAA | AAAAGGGTAATCGTTAGAAACAGAA |
| SSR06913 | TCAAGGTCTCTTATGGGTTGC | TTTCCTGCACATTCCTTTCC |
| SSR10874 | CTGGTTATAAATTCTGATGGTGATT | ATGCTGCCATGTTACTCGTG |
| SSR17108 | TTTCAAACCTTCTTTTAAAATGGC | TGGATGCACTCAACTGAAAA |
| SSR16462 | TCACCTTCATGATTTTGCCA | GTCTTCAACGCAACCCAAGT |
| SSR03606 | TGGACGAGACCTATCAAGGAA | TTTTGGATGGACCTTACCCA |
| SSR12682 | ATCCAACCCAATCCAAATCA | GTTGTGAGCTTAAGGGCGAG |
| SSR05210 | TTTATGCCATTGTGCGTGAT | TCAAATCAACGTCAACCCAA |
| SSR25694 | TCATGAACTGAGTGTTAGATTTGGA | GGGGATTTCATCATCGACAC |
| SSR11343 | GTGGGGTTGCTTTTGGATAA | CAATGGTTGCTTTGCTTCAA |
| SSR20852 | AATGGAGCCAAAAATGTGGT | CCAAGCAATTGAATTTTCCC |
| SSR01903 | CATTGTCCTGATGGGGAAAG | ACCCTTTCTCAGCGGCTATT |

| Name | Forward Primer Sequence | Reverse Primer Sequence |
|---|---|---|
| SSR23037 | GGGTAATGATCCAAATACCAAA | CCTTCCCTCATCTGTTGTTTT |
| SSR14949 | CGACTTGCTCAACTCGGTCT | TCCTTCCTCCCTTCGTTAAAA |
| SSR07291 | CCAAGGAGAAAGAAGGGCTC | AATCCAATCCAAAATGCACC |
| SSR14290 | TGAAACAAAATTCGAGGTGTGA | TCACCACATTCTTTTTGGCA |
| SSR01331 | CGGGATTTACCCCTCACATT | GTGGGACCGAGAAGTTTGAT |
| SSR33694 | TGAAAATTGGATGAAGGGGA | GCTTTTGCTTGTTCTTTGGG |
| SSR17023 | CCCACAATTTTAATTGACTCCAA | AATTACCCAATCCAGCCATTC |
| SSR00973 | TTGGGGCTGTTCTAATTTCG | TCGTTGTTGAAGCCAAAGAA |
| SSR20859 | CACTGATTGATCCCATGTGC | TCGCTGCATATCTTCCTGTG |
| SSR32935 | TTCTGTGGTTCATCTGGACG | CACACCAACCAGAATCATCAA |
| SSR19174 | TGCCGTACACAAATTTCAAATAC | GAAGTATAATATGCACAAATCCCA |
| SSR05949 | TGTCTAACTATTGCTTCCCCG | GGACTCGACCCTTCGATTTT |
| SSR11985 | GCTGCATTTCATTTAACGCTT | TGGTCCATCCTCACCAATTT |
| SSR18289 | TGGTTTTGAAGACGGAACAA | CCACCCACCCTTACACAGTA |
| SSR19165 | AATCCACGTTGGTTGTCGTT | GAAGGGCCAAAAATGTTTCA |
| SSR30097 | AAAGCAATTCTCATGTTTTCCC | TTCGTCCTATTTGTACGACGTG |
| SSR15482 | AAAAACAGCAGGGCAGAAGA | GTTGAGCTGTCTATGGGGGA |
| SSR13611 | ACGGGAACACTCATGTCTCA | TTTCGTTGTTTAGTAGACGTTAAAAGA |
| SSR00233 | AACCATAAAGTCGGGAGGGT | GGGAAAGGCAGGAGAAAAAC |
| SSR01234 | TTGCTTGGTAAGAAGAGTTGAGG | ATGGGTGCAAATAGCAGCTT |
| SSR11244 | TGTGGGACCCTCCAATTAAG | AGATTTTGTCGCCACGTGAT |
| SSR14001 | GGCTTGATTTTCGGTTACGA | CTTTTTCCGGTACACATCCG |
| SSR01643 | TGCAGGTCGACAATTCAATAA | TCAAAAGGCACATGTGATGTC |
| SSR18251 | AAATGGCAAAATAATGCATGG | GAGACAGCCACAGAGATTTGG |
| SSR05946 | CCTGAGAATCGAAGGTCACA | GCCATCACTAACTGACGCCT |
| SSR01873 | TTTTCTCTCCCACGTTTGTTG | CAAGTGGGCTCGGTTCACTA |
| SSR00193 | GCCAATCCAATGGAACAAGT | TTGTAAACCAAAACCTTACCCC |
| SSR11512 | CCAAAAATCTTGCATTTTTAGATCA | GGGTAATTCCCCATTGGTCA |
| SSR21440 | TTGTATCAAATTGTGCCTTAAAAGTT | TGCATGCTTATCTCCTTTTCA |
| SSR12347 | TTCGAATTGGATTGGGTCTC | GCAATCGGTTGACTTTCCTT |
| SSR07108 | TAAGCAATTCCAGGAGAGGG | GTTCTTTGATGGGTGCCTGT |
| SSR10518 | TCTAATTCGCTCCGGATGAT | TTGCAGCGAACAATCCTGTA |
| SSR01286 | CCGAAAACCATTGTTCAAGC | TTTAGCTTAGTTTCCAAGCACTGA |
| SSR15172 | GGTGTGGGTTATTTTGGCAC | GAAGAAATCAAAGAGGGGGC |
| SSR17814 | TGTTGCTTACCCAAAAAGGG | TTGGCATTATGTGATGATTTGA |
| SSR13818 | TTGTTAGTTCATTTGAGGTGTCAAG | TCCATATTAACTCTCTCAGGCTAACA |
| SSR12083 | GAATTGGCCCATCCTTCATT | GCCATTCCAAAAACTTTTCAAC |
| SSR05865 | AGCCAAGACAATTCACAGCC | TTTCCTATCGGGTCTTCGTG |
| SSR16941 | ATCGGTGGTAGTGGTTACGC | GTGGGGTCCAGAGTTGAAAA |
| SSR22227 | ACGTGAGAGACACCCCTCC | TATCCCATGGGTATGGTGTG |
| SSR23732 | CCACCTCACATCTATGGGCT | GATAAGACCACATGGTGGCA |
| SSR05492 | GCAACCATTCTTTACTGTGCC | AGGGCATTTCAAGACAAACC |
| SSR13275 | CACCTTGTTTCGCACGAGTA | GGAAAGGGACCACAAATTCA |

| Name | Forward Primer Sequence | Reverse Primer Sequence |
|---|---|---|
| SSR23757 | TTCGCCTTTACTTCCTTCCA | CGGCAAAATTCCTCCATTTA |

**Table S2.** The sequence of all CAPS primers used in fine mapping of the white immature fruit skin color trait

| Name | Forward Primer Sequence | Reverse Primer Sequence | Restriction Enzyme |
|---|---|---|---|
| H1-1 | AGAGGAGGTCAGCTCCATGA | TGAAGCGTCAAGATGTGGAG | XbaI |
| H1-2 | CTCTAACTCGAGGGGCAGTG | CCACCACTGAATTTGCCTTT | MspI |
| H1-3 | CAATTCCTGCGGAGTTTCAT | GGGTGGTTTGAAGCATAGGA | MseI |
| H1-4 | TTGGTGGCCCATCATAATTT | GATACTGGCGGTTGCGTAAT | MspI |
| H2-1 | ACTGGCTTTGAAGACGAGGA | GGTGCAGCTCAAGGATTAGC | KpnI |
| H2-2 | CCACTCCCTCCAAAAGAACA | TTGCAAAGCTCAACCAACTG | HindIII |
| H2-3 | TATCATCGCCATCAGATCCA | CTTTTGTGTGGTGCATTTGG | EcorV |
| H2-4 | ACACGTGGCAAAAATGAACA | ATTCATCGTTGTGGTCGTCA | EcorI |
| H2-5 | TCGAACCACTTGAGCTTCCT | GTAATGCTCAATGCCCCACT | EcorI |
| H3-1 | CCAAACCCCTCCAATGTATG | AACATGCACCACAACCAGAA | EcorI |
| H3-2 | AGATTGGTCCTCCGTGTGAC | GTGCCCACTAACCTTTGCAT | MspI |
| H3-3 | CGACACATTGATCCGAACAC | CTTTGGGAAATGTTGGCACT | KpnI |
| H3-4 | GTCGGTTTTCGTCGATGTTT | TGTGCCAGAGATTGCTGAAC | RsaI |
| H3-5 | CACACTTCAACAGCGAAGGA | AGCTTTGATGGAGGCTGAAA | EcorV |
| H4-1 | GTGACCCGAAAGTGGAGTGT | CGGATCCAGTTGTTGGAGTT | EcorV |
| H4-2 | GTGAAAAGGCAGCTGAAAGG | GAAACCAACAAGCACGGAAT | MspI |
| H4-3 | GTTGGTGAGGGTTGTTGCTT | GGGTCAGACGATGGAACAGT | EcorV |
| H4-4 | TTTTGGCTATTCTCCCATGC | CGTGCTCTCAATTGGGAAAT | MseI |
| Q1-1 | GCAATGCAAAAGCAGTCAAA | TTGTGGCAACACAAATCGTT | MspI |
| Q1-2 | GGCCTTATTTTCCCTTTTGG | AGAAGCTGGGAGAACGTGAA | EcorI |
| Q1-3 | GTTGCAAAAGTGGAGGAGGA | CATCCCCTTCTCCAAGATCA | XbaI |
| Q1-4 | GAGGGACTGCCACGTATGAT | CTCGGTGTCGGTAACAACCT | HindIII |
| Q2-1 | TTAACCCACACGGCAGTACA | CAACGCCAATGCATTTACAC | XbaI |
| Q2-2 | TTTCCAAGCTGACCCATTTC | TTGTGTCGTGCACTCATTCA | MspI |
| Q2-3 | GCCATAATGCTTTTGTGCAG | TTCACGATGAAACCGCTACA | HindIII |
| Q2-4 | ACGAGACGACCGATCAATTC | TATGGTGTTCGCTCTTGCTG | MspI |
| Q3-1 | GAGGATTCATGCCTTTTGGA | GCAGAATCGGGACAATGAAT | MspI |
| Q3-2 | GGGATCGAAAATACCCGAAT | TTTATTCCCGCATCGAAGAC | HindIII |
| Q3-3 | TGCAAGTAGTCCCTCCAAGTG | CTGCATGCTCTGTTCTCACC | HindIII |
| Q3-4 | CCAAGTCCAAAAGGGATGAA | CTTCCAAATGGCCAAGGTAA | MspI |
| Q3-5 | TGAGGAACCCACACAATGAA | AATCCCCAAGAATTCGATCC | XbaI |
| Q4-1 | ATAAACGCGGTGAAAGGATG | TGGAAAAGGAGAGGGAGGTT | EcorV |
| Q4-2 | GGATCTTCCGGGGACATTAT | TAACCACAACCAATGGCAGA | MseI |
| Q4-3 | GCTCATGGTGTACGTTGTGG | AGATACGCTCACCTGCCTGT | EcorI |
| Q4-4 | TCTTGCTGAGCCAACTGCTA | CTTTCCCACTCTCCAAGCTG | MseI |
| Q5-1 | GTCACCTCTTTACCCCACGA | TCAGAGCCATCTTGTTGCAC | HindIII |
| Q5-2 | GACTCGAGGCCATCTTTCAG | TGACGACAAAGGGTTGAACA | MseI |
| Q5-3 | TGGCTCCTTTCCTCTGACAC | TTGAAACCCACACAAAGCAC | EcorI |

| Name | Forward Primer Sequence | Reverse Primer Sequence | Restriction Enzyme |
|---|---|---|---|
| Q5-4 | CCATGGGTGGTTCTTTGTCT | ATTGCCTCCTCCCATCTTTT | EcorI |
| Q400-1 | GAAGGACCATTTGACCGAGA | TTACGTTCGCAGAGATGTCG | EcorI |
| Q400-2 | CGACTTTTCAATGTCCACTCC | TCTCCATCGGCTTTGATTTC | MspI |
| Q400-3 | TGCTGCATTTTGTTCAGAGG | CCTATGTGCAACCACCACAG | KpnI |
| Q400-4 | CATTTGCAATTCCACGAAGA | GTTGTTGTCATGTCGCATCC | HindIII |
| Q400-5 | TACAACTTCCTGCCCCACTT | TTCAATCCTCCATCCCTCTG | EcorI |
| Q400-6 | GATTGCCAAGCCACGTTTAT | GCGGGATTCAAGAAACAAGA | EcorI |
| Q400-7 | CTACCCAGCTTGGCCTTGTA | GTTGTTCCTCTGAGGCTTCG | XbaI |
| Q400-8 | GGCTCTGCTAGGTCAACGTC | GGCCAAACACAGAGAAAAGG | MseI |
| Q400-9 | TTCAAACTCAGCTTGGTCAAAA | ACCCGAGCTTGATTAAATGG | EcorI |
| Q400-10 | TCTCACCCTCATTTGCTTCC | CGAACCCTTCATCATCAGGT | MspI |
| Q400-11 | TCTCTCCTTCCCCTTTCCTC | GAATCCCACTTGCCAGAGAA | MseI |
| Q400-12 | GGCAGACCAGAAAGACAAGC | GCGTAGGAAGCTGATGGAAG | MseI |
| Q400-13 | GGGATAACTCCATGCCAATG | AAAAAGGAGGAGCGGCTTAG | MseI |
| Q400-14 | TCTCTCGCCACCAATTATCC | ATTCCTTGCACGAAGTTGCT | RsaI |
| Q400-15 | AAACCATCAGGAACGTCTGC | GAGTCCCTCCACCTGTACCA | RsaI |
| Q400-16 | ATGACCGGACACTCTCGTTT | CAAGCCGTCTCCTTGAACAT | XbaI |
| Q400-17 | GCACCAACGGAAATTTGTCT | TCTCGTCGCCTTTTCCTCTA | MspI |
| Q400-18 | TACCCCCAGAGGTTCTGTTG | GCCAGCTCTCTGTTCAATCC | MseI |
| Q400-19 | GACGATTGGCCTGTTTGAAT | CCACGAACAGCCTCTCTAGG | MspI |
| Q400-20 | CAACCCGCCAACCTTTAGTA | GTCCCAAACCTGCAAGTGAT | KpnI |
| Q400-21 | TCGCTACATTCATCCAGCAC | GAGGTTGAGCTTGGTCTTCG | HindIII |
| Q400-22 | TTGTTCGATGAAGTCGCTTG | TTATCATGGGACATCCAGCA | RsaI |
| Q400-23 | GGGAAAAACGAATGTCCAGA | TGTGCCACATTCCAATTGAT | XbaI |
| Q400-24 | CTTGCAGGAAGGTTCTCCAG | CAATCCAAACAGGCCTTCAT | EcorV |
| Q400-25 | GGAAATGTGTGGAGCTGTCA | ATATCTGGCCCTTGCCTTTT | MseI |
| Q400-26 | TGCTTGGCCAATAGTACGTG | ACGACACCTACCCAATCTGC | EcorI |
| Q400-27 | GATGGAAATGGGAGCACAGT | GCAAATGGAGCAGTTTGGAT | MseI |
| Q400-28 | AAGTTCTCCAATCACGCCTTT | CCCATCCAACACTTTCATCC | HindIII |
| Q400-29 | TGCAATTCTTTTCCTTCAACAA | ACTTCCTTTGGCCCATCTCT | MseI |
| Q400-30 | CCCCAATGAAAGACCTTTGG | CCAATTCCACGAATTTGTCC | EcorI |
| Q400-31 | AGGCATCGTACAACCCAGTC | GGGTTTTCCCAATCTGTGTG | Tsp509I |
| Q400-32 | CCCCTCTTTCTCCTCCCTTT | TTGAATGCAACAAAGGGTCA | EcorI |
| Q400-33 | CGACGTGCCTTACCATACCT | ACTCCCTAGGTAGCCCCAAA | EcorI |
| Q400-34 | CAGCACAGCAACGCATAGTT | CCCCCTTCAACCTTGTTTTT | MspI |
| Q400-35 | TTGGATGAGCTCTGCCTTAAA | CGAAAAAGGGAAAGGGAAAG | MseI |
| Q400-36 | TTGTCAAGGGCGATGTAAAA | CACGAGAGAATGTTGGGAAAA | MseI |
| Q400-37 | TAAAATGGGCGCTTTTGAAT | TGTGGCATCATTTGGTTTGT | XbaI |
| Q400-38 | GCACAAACCTCCTCCTGCTA | TGACAGGGATCACAAATCCA | XbaI |
| Q400-39 | CGGATTTGGAGCTGAAGAAG | ACATTTGACCAACGCCTTTC | XbaI |
| Q41 | TATCTCCCTCACCGAACCTG | ATGGAACCAGTTGGCTGAAC | MseI |
| Q42 | GCATTAGATCCGGGAAACAA | ACAATCCTTGGCAGAGCAAT | MspI |
| Q43 | AAGAAGCTGCTGCTCTGTCTG | CTCGAGCTCATGGATGTCAA | HindIII |

| Name | Forward Primer Sequence | Reverse Primer Sequence | Restriction Enzyme |
|---|---|---|---|
| Q44 | TGGAAAATCCGTTCGTCTTC | TCAAAGTCCAATTCGTCGTG | HindIII |
| Q45 | GATGCCAAAGGGATTTCAGA | CTTGCTCGTTGGATCACTCA | HindIII |
| Q46 | AACGGCTGCTGAAACTGAAG | AGGAGTCATCAAAGCCATGC | MspI |
| Q47 | TTGATGCGCATGTATTGGTT | ATGGGATGATGAATGCCAAT | EcorI |
| Q48 | GATATCGCCAGTCTCCTCCA | GGTGAAGACGGTGATGTTGA | EcorI |
| Q49 | TCACCACATCAATTCCTCCA | GGAAAGGGTGGCAGTGTAGT | HindIII |
| Q50 | AGCATAGCAACAGGCCAAGT | CATAAGCTCGATGGGATCGT | HindIII |
| Q51 | ATGATAAAAGGGCGTCATGC | CGCTGATGCATTAAGCTGAT | EcorI |
| Q52 | AGCTGGATCTGCTTCAGTTTG | ATCGCATATTGCTGGATGTG | EcorI |
| Q53 | TCCTCACCTTCCTCAGCCTA | GGTAGTGGAGGCAAGAGCAG | RsaI |
| Q54 | CCAGAAAGGCAAGCAAAAGA | CCTTCATGGTTTGGCAGATT | MseI |
| Q55 | TTACTTGGGTTCCAGGCAAG | CTCCTCACTGCCAAAACCTC | EcorV |
| Q56 | AGATTGCTGCAGGTTCCATT | GCCAGCTCCTTCTCACCTAT | HindIII |
| Q57 | TGCTCCCAAAGTTGCTAAGG | TTTTTGCTGCTTGCCTTTCT | MseI |
| Q58 | CCAGGCCCACTATCTCTTCA | ACCAACCAAGGTCAAGATCG | EcorI |
| Q59 | CTGGCAAGGCTTTGGTATGT | ATCCTACAGCCTGCCTCAGA | MseI |
| Q60 | GCAAGTCCCAAACAAAGAGC | CTCGATGGAAAAACCCAGAA | KpnI |
| Q61 | AATTCCATTCCCCTTTCTGC | AGCTTTGTCGGGAACATTTG | HindIII |
| Q62 | TCCGTAGGTCTTGAAGCACA | TGCTGCTTTTCTTGCTTTGA | EcorV |
| Q63 | TTGGGATTTTGGAGGATGAG | TACTCAATTGCATCCGCAAG | EcorI |
| Q64 | GCTCTCTAGCCGATGCAAAG | TCCGTTTCATGGATCTCTCC | EcorI |
| Q65 | TTCTTCGAGCACCTCGATCT | CATTGCAGAGTCTTCCAGGT | EcorI |
| Q66 | TCAGGCTTTAACTGCCAAGG | CTGATGTCGGAAGCTGGTTT | MspI |
| Q67 | GGATGCCTTACCTTCTGTGG | AAGCCAATTGATGCTCCAAG | KpnI |
| Q68 | TGGATGACATGGTAGCTGGA | GGCGGCCATACTTTATACCA | RsaI |
| Q69 | GATGGGGAATTGCTGAAGAG | CTTTTGTGCCAATTTCAACG | RsaI |
| Q70 | CTATCCGACATGGCAAACCT | TGCTTCGGAGAGCACCTTAG | HindIII |
| Q71 | AACAGTTTGCGTCCTTGTGA | ATTTGATGCCCTCCAAACAG | HindIII |
| Q72 | TCGACTAGGCGTTCATTCCT | CAGTGGAAACTGCCCTCTTC | HindIII |
| Q73 | TCGAACACGAACGAAGTTTG | TTTTCCACACACATGCTCAA | HindIII |
| Q74 | TGTCTCACATCCCCATCTCA | TGGGTTGATTTGGTGACAAG | XbaI |
| Q75 | AAGACGAACACGCCTCAAAT | ATTGCGAGAGGAAAATGGAA | XbaI |
| Q76 | TCATCCCCTGGTTTATGGAA | GTACGGCCGTGGAATAAGAA | HindIII |
| Q77 | AAAGCCAATCATCCACAACC | AAAAGGCCAACAAGCAGAGA | MseI |
| Q78 | ACCTCAAATTTCCCCAGCTT | ACTGAGCCACTTTTGGATGG | MseI |
| Q79 | AAGGTCTTCGAAGCAGTGGA | CAGCTGTGATGGCTGTCAAT | MseI |
| Q80 | CAAAGGAAGGAGCAATGGAA | GAATCAGGAACCCACTTGGA | HindIII |
| Q81 | TGCAATCAATGCAAGAGGAG | TCGGGGACCAGAGTGTTAGT | HindIII |
| Q82 | AAACCTTCTTCCATGGCTCA | TGCTTCTTGTCGATCCCATA | RsaI |
| Q83 | GTGGTCCGTGGTCTGCTAAT | AAGACGGGTGCAACTGAATC | MseI |
| Q84 | GGGAAGGATATCCGCAAAAT | GGCCACTGAACAGGTGTTTT | EcorV |
| Q85 | TTTTTCTGGCCAGGTGCTAC | CAGCCGAGGAAAAACAGGTA | EcorV |
| Q86 | AATTCGACGAGCAGACAGGT | AGAAATCCACCCTTCCAACC | MspI |

| Name | Forward Primer Sequence | Reverse Primer Sequence | Restriction Enzyme |
|---|---|---|---|
| Q87 | ACCACCAAAGGTTCCAACTG | CCCCGTTGTTAAGGTATGGA | EcorV |
| Q88 | CCCACTCTTTTGCCTTAGCA | TTGAGTCACGAACTCCTTGG | MseI |
| Q89 | GCGTCTAAACCTGCTCGTTC | AAGGGTGTGACCATTGAAGC | MspI |
| Q90 | GGCTCATCGAAGACTGGAAC | GGTTCCATTTGAGCCACATT | EcorI |
| Q91 | AGGGGTGCAATGAATGTGAT | CTTGTGCCACCTTTGTTTCA | XbaI |
| Q92 | CCATGGTTTTCTCGAGTTGG | CTACTTCACAGCCCCACACA | HindIII |
| Q93 | CGACGGAGTAGAACACTACACG | TATGCACCAATCCCACAAAA | XbaI |
| Q94 | AAGCAATTGCAACCCGTAGT | GATCGTTCTGCATCCGAGTT | XbaI |
| Q95 | TGCCGATTCTTTTCAAATCA | TCATTTCCACTCCCAACCAT | HindIII |
| Q96 | CGGATCCCACCTTGTTTAAT | AGGGCAAAGGCAACTATCAA | HindIII |
| Q97 | ATGTGACCCAGGAATGGTGT | TTTTCTGCTGGGCTTGATCT | EcorV |
| Q98 | AAGACGCGAGGAACAGAAAA | ATCCTCAAGGTTCCAGCAGA | XbaI |
| Q99 | ATGTAAGGGGCAAATCCTGA | GAATCGTCACGACCCTTTGT | EcorI |
| Q100 | TCCCACACATCAATCAGAGC | GGCGAGATCGAGAGAGTGAC | MseI |
| Q101 | GTATGCCCTCTGCTGCTCTC | ATCTGTTGCAGGTGTTGCTG | EcorI |
| Q102 | GTTTTTCATGACGGCTCGAT | GGCAAAATTTGTCGAAAGGA | MseI |
| Q103 | CCTTCTCGGATGCTTTTCAG | AGCTTATGTGGCGCTTGTTT | HindIII |
| Q104 | TTGCAAGTTGCTGTGTTTCC | TTGGGGTTTCCCCTTAGTTC | HindIII |
| Q105 | AAACCCATCGATGACAAAGG | GCTTGTAACGGGGACGTAGA | XbaI |
| Q106 | TGGTTCAAATAGCCGTTGCT | CACCATCTCGAATGTCTTGC | EcorV |
| Q107 | GGCGAGCAGTATGTCTGTGA | CAATGTGGGTGTCTTTGGTTT | MseI |
| Q108 | CCGGACTTAGGGACTTCACA | TCGCTCCCGTATCAACCTTA | MseI |
| Q109 | CCAACTCCAAACACACACCA | TCAACTCGATTTCCCGTCTT | HindIII |
| Q110 | CCCAACATTGCGAGTAACCT | GGTTGACAAGTGGTCGGAAT | MseI |
| Q111 | TTCACGGGAAAAATTCCAAG | GCTTCCACCGTCATCAATTT | EcorI |
| Q112 | TTGCCAAATCCCATTTGTTT | TCGGAATTGGTCATTACTCG | HindIII |
| Q113 | GTCGGGGAAGCAATGTAAGA | TATGCGAGTGGCACGTTATC | EcorI |
| Q114 | TACAATTTGGGGTGGGAGAA | TGGTCCGAAGTAGGTGTGAA | EcorI |
| Q115 | CTGCCATGCTGAAAGAACAA | TTTTGGCTCAATGCACTCAA | XbaI |
| Q116 | GGAGCCTCCATGCAAACTAT | TTGCCCTGACCCTACTCTTG | MseI |
| Q117 | TAGTCCATTTGTGCCTGTCG | CACAGCACACACACACGTTC | EcorI |
| Q118 | TGAGGGTGCATCTAGCAGTG | GGAAGACCCACAAGGAGACA | EcorI |
| Q119 | TGCATGCAGGTGCAATAAAT | CATTGTGCCCGAGAATAGGT | XbaI |
| Q120 | TCTGGTTGTGGGTTGTCAAA | TGTGAAGCAATTACGCAAGG | MspI |
| Q121 | GGCTAGCCAGGGTGTCAATA | CGTCACATATCGAATTTAAAGCA | HindIII |
| Q122 | TCCCCTACTTCCCTCCTGAT | AAGAGTCGGACCAGCACAAT | MspI |
| Q123 | TTGTTGCAAGGAGCACAATC | AATTCCAAACAGTGCCAAGG | MspI |
| Q124 | ATATGCACCCAGAGGTGAGC | ACAATCCAAGGGTGTTCGAG | HindIII |
| Q125 | CTGGCATGTATGTTTGATGGA | CACCAAAGCTTCCACTTGGT | HindIII |
| Q126 | TCATTCCCATGCTTTCTTCA | TTTCTCGCTTCATGGGATCT | MspI |
| Q127 | ATACAAGCAAGGCCAACCAC | ATAAGAAATGGGCCGGAACT | EcorV |
| Q128 | AAGGAAGGCGTCAACTAGCA | CCATTCCTCTCTGGGATTCA | HindIII |
| Q132 | GCAAGAAGCAACAACATCCA | GGGAAATGGCCCAATAAAGT | EcorI |

| Name | Forward Primer Sequence | Reverse Primer Sequence | Restriction Enzyme |
|---|---|---|---|
| Q133 | AAAAAGAAACCCCACGCTCT | TGAGTTTCATGCGAGATTGC | MseI |
| Q134 | AAGTGCCTGAACCAATGGAC | AGTTGGACCAGCACCTTGAC | HindIII |
| Q135 | GCTTGGGGTAGGAGATAGGC | AAAATTGGGGGTGGCTTAAC | MseI |
| Q136 | TTCCTTTCGCACCGTTAATC | TTTGACATGGCACCTGATGT | EcorI |
| Q137 | GTTGGGAAGAGGGTGTCTGA | AGAATGATCGGCTTCAATGG | EcorI |
| Q138 | TAGGTTAGGCCAGGGACATC | TTTAAGGCTGGTGGTGATCC | RsaI |
| Q139 | GAGCTCCACTTTGGACAAGC | GAGCTCCACTTTGGACAAGC | MseI |
| Q140 | CCTTGGAGCATCTAGGCAAG | GACATGTGGAACATGCTTGG | HindIII |
| Q141 | AAGCTGATACATGGGGTTGC | TGGCTTCTTCATTGCCTCTT | HinfI |
| Q142 | AAGCTGATACATGGGGTTGC | GGTGACCATGGCTTCTTCAT | MseI |
| Q143 | TACTCACGCCCCAAAGAAAC | AAGCGCCAAGCAACTTAAAA | EcorI |
| Q144 | CCTTGGAGCATCTAGGCAAG | GCTTGGGACACTTCTTCGAC | EcorI |
| Q145 | TTGGAGAACGAGAGCAACCC | CCGATCTCAACTTGGAATATGGA | XbaI |
| Q146 | TTTCCTCTCATAAGAATTTTGGACG | ACTCACACTGGTTGGAACTCT | HindIII |
| Q147 | AGGCCGACCATCATCGTAAC | ACTGGAGTGCCCTAGATGCT | HinfI |
| Q148 | TGCTTGTGATATACATCTTGGGTTG | ACGTCGTAACGCTAAAACAAGT | DraI |
| Q149 | TGGAGAAATCCTTTCGGACTCT | GGAGCTTGAGCCTTCACAGT | XbaI |
| Q150 | ATACGGAGCATGAGAAGGCG | AGTGTAAACTCTTTGATGTGGACC | DraI |
| Q151 | TAAAATTTGCTGTGGCTGGGC | TGGCAAAATGAGTTTCAACCTTC | EcorI |
| Q152 | TTGGAGAACGAGAGCAACCC | CCGATCTCAACTTGGAATATGGA | HinfI |
| Q153 | AAGGCCCCAAATCATCTCTCTT | CATGTCTCTCTCCCTCAGCG | TaqI |
| Q154 | CAAAGACTCCATGGGACTGGA | GGCAAGTTAGGCAGACCCAG | TaqI |
| Q155 | CCTGACAAGGGTTTTGCAGC | GAGAGTCCGAAAGGATTTCTCCA | HindIII |
| Q156 | GCCAAGACGACTTGTTCAACC | ACAACCACTCAAAACAAACTTCG | AluI |
| Q157 | TTCGAAGTTTGTTTTGAGTGGTTG | CCGCCAAACATGCCATTTCA | AluI |
| Q158 | TGGCATGTTTGGCGGTTTTC | GACCACCGCTAAGGGAAGAG | HindIII |
| Q159 | TTAGCGGTGGTCACACTTCC | AATGTGAAAAACGACCATAGCAGT | TaqI |
| Q160 | TACTGCTATGGTCGTTTTTCACA | GAAGCCAAGAGGTCTCCGAC | TaqI |
| Q161 | TTCCTTCCGCTCAGACCAAC | ACTTCAAATCCCCCACGACC | MspI |
| Q162 | GACAGCGCCAAGCGATATTC | ACTGAAAATCTCCGGCCTCA | EcorV |
| Q163 | GTCCTGGTGAATGGGTGAGG | GAATGAACTGCCGACTCCCA | HindIII |
| Q164 | TAAGCTCGTCTTTCTCGGCG | ACTGGGTTACAGCTTGGTGC | EcorI |
| Q165 | GCACCAAGCTGTAACCCAGT | CCTTGAGTGGAAGCAGGCAT | MseI |
| Q166 | AGAAGCTAAGTGACAAGCAACG | GAGAACTCCCTACATCCCTTCA | HindIII |
| Q167 | AAGTGGATTGAGGAGGTGCG | AACGTAGATTCAGGTTGGAGGT | MseI |
| Q168 | AGCCAAAGCCCGTGAACTAA | ACCATTTCAAAGAACAAGGCAG | EcorI |
| Q169 | TCAAACCCTCCTGCAGTTCG | CCGCGATTTCGATCCAAGAAG | TaqI |
| Q170 | TGACTTCTCTTTCAGTTGTCAAGC | ACTGTCCTATGTGCCAGTGC | DnpI |
| Q171 | ATCTGGAACGGGGAATCTGG | ACTGCAGTGCCATCACTATCAT | DdeI |
| Q172 | TGGCACTGCAGTTTTAAGCAC | CCCGCCTGCAGCAGATATAG | EcorV |
| Q173 | TGGCGCTCTCATTGTAGCAT | ACGTCACTCAATACCTGGACTC | HindIII |
| Q174 | ACTGTGGAGGCAGCATTGTT | AAGTTTTCGTGCTCCTTGCG | EcorI |
| Q175 | GATGGATGTTCTTGCTGGCG | TGCACTTGAACCGGAAGTCT | EcorI |

| Name | Forward Primer Sequence | Reverse Primer Sequence | Restriction Enzyme |
|---|---|---|---|
| Q176 | ACGGAGCTAGACACTATCGTT | ACTCCAAAATGTGAAGACGGC | XbaI |
| Q177 | ACGATCAAAGGGCAACGACT | TGAAGTTGCAAAGCATCACCA | HindIII |
| Q178 | GAATTGGTGCTCACACACGC | GCCGCACTTCTTGAGTACCT | HinfI |
| Q179 | TCCCATGAGAAGGTTGTGTTTGA | AGAGCTCAGAGTACACAAGGT | DraI |
| Q180 | AGAGAGCTGTAAAGCACAATAATTC | CAGGCCATGATAAACTGCATGA | XbaI |
| Q181 | GCTCAGCCTTTTGCTTACTTCA | TACACCAGTCAACGCAAGTT | DraI |
| Q182 | TGGCCATCAAATTGCGGTTC | GCCGATCAACTCCTCCTGTC | EcorI |
| Q183 | GACAGGAGGAGTTGATCGGC | GGTCTGAAAGAAACCAGCGG | HinfI |
| Q184 | ACATAAGCCTTAATTGGAAGGCA | TGCAAGATTTCCAAGGCCAAC | TaqI |
| Q185 | AGCCAAGGTTGGAGAACGAG | GTTGACACCCCATAGCGACT | AvaI |
| Q186 | AGCTAAGAATCAGTGGGCACC | GAAGATGCCAGGGCTCTCTA | XhoI |
| Q187 | GAGGCCTTCGAATTTCATGAGC | CTCAGTTTCGACGAGGGTGT | MluI |
| Q188 | TACGCTCACTGTTGATCGCT | GGTTCGAATCCCCTTACATGGT | EcorV |
| Q189 | ACCCTTCTCGATTGTGCCTC | AGCTCTGATCAAACTGCGTGA | HindIII |
| Q190 | AGGGAGGATGCTACAAGTGA | TCCCTATCCCCTTTTCGGTT | EcorI |
| Q191 | TTCACCGTGGTGGATACAGG | AGGTGGTGTCGTGTAGTTTC | EcorI |
| Q192 | TGCCTCTGCTTCTCCTCTTCA | TCAGACAGCGAGGTATTGCT | XbaI |
| Q193 | AGCTTCAGAGTGCTTCGTGT | GCAGCCTTTTCTCGTTCAACT | Hpy166I |
| Q194 | TCTGTCGCTATCTTGCATCACA | CAATCCGCAGCTATTCGATGG | MspI |
| Q195 | GGATAGATGCAGCGTTTCTGT | TGATTGAGACAGGCCAGCAT | EcorV |
| Q196 | TGGTCCGTACTTATATCGAGAAG | GGGAGCAATCTCAATGAATCACG | HindIII |
| Q197 | CACCCTTGGGTTGGAGTTGT | ATCCTTGTCCAGCTGTGTGG | EcorI |
| Q198 | TTTTCTTTAAATCTCAGGCGACC | CCTCGGTGTACCTGTCTGTG | MseI |
| Q199 | TGGCAGAAACTCAGGTTCCA | AGCTAGCAAACTAAGGGAGCA | HindIII |
| Q200 | GAGAGTGCCACGTGTAACGA | TCAAACGGTGCCGTATGGAG | MseI |
| Q201 | AATTCAAAATGTAAGACCACAGACA | AGCCACTCTGTTCCACATGA | TaqI |
| Q202 | AGATCGACACTTTTGAGACACT | GCCTTCCAGTCACGAGGAAT | AvaI |

**Table S3.** qRT-PCR primers of all 13 candidate genes.

| Name | Forward Primer Sequence | Reverse Primer Sequence |
|---|---|---|
| q4070 | CCTGACAAGGGTTTTGCAGC | TCACTGAGGAAAGCCCCAAC |
| q4080 | CCATCGAGTGTCCACAAGAGC | TTCGTCATCAGTGAAGGCAAA |
| q4090 | ATCGTCGTTTCACTCGGCTT | TCATGGCCGTGTCGATTTCA |
| q4100 | CACCCATGCTGTTTTCAGCC | CGTGAACACGCCAAATGGTT |
| q4110 | TACGATCCACGTCAAATCTCCTT | TGCTTTCTAGTCCCTTCTCAACA |
| q4120 | TCCTCGAATGGCTGCTTCTC | CTCACCATCTCGGCAGTGTT |
| q4130 | ATGATGCTTGGAGTACACGAATG | CAACTTGCTGAAACCTGTGAGAC |
| q4140 | TCAGGAGGGCCAACCTCAAT | GGGTCCAGTCCACCTTTGTT |
| q5140 | CTCATGGTGTACGTTGTGGC | GGCATTGAGACACCACACCT |
| q0640 | TTTCTTACTGCTTGTGGGATGC | TGACCGCTGTTTCTAGTTGACG |
| q0650 | ATCCGACTCCACTTCCAAGC | AGCCGAACTCGAAGATGGTG |
| q0660 | TTGAGGAAGCGGAATGGGAC | TATCTCCCACCGTCTCGGTT |
| q0670 | AACAGATCAAGGCTCAGGTCC | CATCAAAGAAACAGCGGCATA |

**Table S4.** Primers used in TA cloning.

| Name | Forward Primer Sequence | Reverse Primer Sequence |
|---|---|---|
| CDS904080 | ATGGCGACGTTTAATCTCTC | TCAACCTTGGATCCTCCGTA |
| CDS904110 | ATGGCGACAGCTTCCCCATC | CTAGTCCACATCTTTCGGTTG |
| CDS904130 | ATGGCCTGCAGAGGGTGCTT | TCACATGATTGTGCAGCGGC |
| CDS904140 | ATGGTTTGCACTGCCGACGA | TCAGGGAGATCTGGAACCGT |

**Table S5.** The list of 17 green/white germplasms

| Material Code | Fruit Skin Color | Origin |
|---|---|---|
| HWB | green | Wuhan, China |
| CC | green | Wuhan, China |
| CD | green | Wuhan, China |
| 2F | green | Wuhan, China |
| 2M | green | Wuhan, China |
| 4A | green | Wuhan, China |
| 4AA | green | Wuhan, China |
| 11 | green | Wuhan, China |
| 1103 | green | Wuhan, China |
| XUAN | green | Wuhan, China |
| PD1 | green | Yuxi, China |
| PD2 | green | Shuangliu, China |
| PD3 | green | Tongjiang, China |
| PD4 | green | Yibin, China |
| DB1 | white | Dayi, China |
| DB2 | white | Qijiang, China |
| BP | white | Lanxi, China |

**Table S6.** The mutations of *Csa3G904140* in two parental lines and 17 green/white germplasms

| Material Code | Fruit Skin Color | G Base Insertion |
|---|---|---|
| Q1 | green | No |
| H4 | white | Yes |
| HWB | green | No |
| CC | green | No |
| CD | green | No |
| 2F | green | No |
| 2M | green | No |
| 4A | green | No |
| 4AA | green | No |
| 11 | green | No |
| 1103 | green | No |
| XUAN | green | No |
| PD1 | green | No |
| PD2 | green | No |

| | | |
|---|---|---|
| PD3 | green | No |
| PD4 | green | No |
| DB1 | white | Yes |
| DB2 | white | Yes |
| BP | white | Yes |

**Table S7.** The 3 SNP mutations of *Csa3G904080* in two parental lines and 17 green/white germplasms

| Material Code | Fruit Skin Color | 3 SNP Mutations |
|---|---|---|
| Q1 | green | T,G,T |
| H4 | white | G,A,C |
| HWB | green | T,G,T |
| CC | green | T,G,T |
| CD | green | G,A,C |
| 2F | green | G,A,C |
| 2M | green | G,A,C |
| 4A | green | G,A,C |
| 4AA | green | G,A,C |
| 11 | green | G,A,C |
| 1103 | green | G,A,C |
| XUAN | green | G,A,C |
| PD1 | green | G,A,C |
| PD2 | green | G,A,C |
| PD3 | green | G,A,C |
| PD4 | green | G,A,C |
| DB1 | white | G,A,C |
| DB2 | white | G,A,C |
| BP | white | G,A,C |
